# Hearing loss impacts neural alpha oscillations under adverse listening conditions

**DOI:** 10.3389/fpsyg.2015.00177

**Published:** 2015-02-19

**Authors:** Eline B. Petersen, Malte Wöstmann, Jonas Obleser, Stefan Stenfelt, Thomas Lunner

**Affiliations:** ^1^Eriksholm Research Centre, SnekkerstenDenmark; ^2^Technical Audiology, Department of Clinical and Experimental Medicine, Linköping University, LinköpingSweden; ^3^Linnaeus Centre HEAD, Swedish Institute for Disability Research, Linköping University, LinköpingSweden; ^4^International Max Planck Research School on Neuroscience of Communication, LeipzigGermany; ^5^Max Planck Research Group “Auditory Cognition”, Max Planck Institute for Human Cognitive and Brain Sciences, LeipzigGermany

**Keywords:** alpha oscillations, hearing loss, hearing aid, cognition, working memory

## Abstract

Degradations in external, acoustic stimulation have long been suspected to increase the load on working memory (WM). One neural signature of WM load is enhanced power of alpha oscillations (6–12 Hz). However, it is unknown to what extent common internal, auditory degradation, that is, hearing impairment, affects the neural mechanisms of WM when audibility has been ensured via amplification. Using an adapted auditory Sternberg paradigm, we varied the orthogonal factors memory load and background noise level, while the electroencephalogram was recorded. In each trial, participants were presented with 2, 4, or 6 spoken digits embedded in one of three different levels of background noise. After a stimulus-free delay interval, participants indicated whether a probe digit had appeared in the sequence of digits. Participants were healthy older adults (62–86 years), with normal to moderately impaired hearing. Importantly, the background noise levels were individually adjusted and participants were wearing hearing aids to equalize audibility across participants. Irrespective of hearing loss (HL), behavioral performance improved with lower memory load and also with lower levels of background noise. Interestingly, the alpha power in the stimulus-free delay interval was dependent on the interplay between task demands (memory load and noise level) and HL; while alpha power increased with HL during low and intermediate levels of memory load and background noise, it dropped for participants with the relatively most severe HL under the highest memory load and background noise level. These findings suggest that adaptive neural mechanisms for coping with adverse listening conditions break down for higher degrees of HL, even when adequate hearing aid amplification is in place.

## INTRODUCTION

Adverse listening conditions are common in everyday life. Auditory distractions and signal degradations increase demands on attention and working memory (WM; Shinn-Cunningham and Best, 2008). WM describes the system for temporary storage and processing of information to perform a cognitive task (Baddeley, 1986). Any degradation of the sensory auditory input requires increased WM involvement to successfully interpret the stimuli (Rönnberg et al., 2008; Stenfelt and Rönnberg, 2009). Auditory stimuli can be degraded by external factors, often occurring in the form of background noise, in which case WM is engaged to extract useful information from the auditory input (Pichora-Fuller, 2003). However, auditory processing can also be disrupted by internal degradation, such as sensorineural hearing loss (HL). To alleviate this internal degradation of the auditory input, people suffering from HL are typically treated with hearing aids. The purpose of a hearing aid is to amplify the auditory input to make sounds audible and consequently reduce the internal auditory degradation, which theoretically should release WM resources (sometimes referred to as lowered cognitive load; Lunner, 2003). Here, we tested whether HL affects brain signatures of WM involvement in an adverse listening paradigm.

The power of neural oscillations in the alpha frequency band (liberally defined as 6–12 Hz) has been found to increase with WM load (Jensen et al., 2002). According to the functional inhibition framework (Klimesch et al., 2007; Jensen and Mazaheri, 2010), alpha oscillations indicate the inhibition of currently task-irrelevant brain regions and/or cognitive processes to prevent interference with task-relevant cognitive processing (Bonnefond and Jensen, 2012). Although alpha power modulations have been found for external degradation of auditory signals (van Dijk et al., 2010; Obleser and Weisz, 2012; Obleser et al., 2012; Becker et al., 2013; Scharinger et al., 2014; Wöstmann et al., 2015), it is currently unknown how the internal degradation of auditory input through HL affects neural alpha dynamics (Strauß et al., 2014). There is good evidence from behavioral studies that HL negatively affects cognitive operations on the speech signal (McCoy et al., 2005; Wingfield et al., 2005, 2006). These findings support the hypothesis put forward by Rabbitt (1991), stating that adverse listening conditions require the allocation of more cognitive resources, which could otherwise be used for more task-relevant cognitive processing, such as storing information. Thus, external (acoustic), and internal (auditory) degradations are assumed to trigger a higher degree of WM involvement during the encoding of task-relevant stimuli, leaving fewer cognitive resources for the storage, and processing of information in the WM (Lunner et al., 2009; Van Engen and Peelle, 2014). Here, we tested whether HL impacts behavioral performance and neural mechanisms even when it is treated with individually fitted hearing aids.

A well-established experimental paradigm to test WM demands is the Sternberg paradigm (Sternberg, 1966). Participant’s task is to encode and retain a number of items to compare them to a subsequent probe. Although the Sternberg paradigm was originally developed as a visual WM task, it has since been adapted to test auditory WM (e.g., Rojas et al., 2000; Leiberg et al., 2006). The test incorporates a short stimulus-free delay period between the encoding and the probe presentation, during which the participants are to retain the presented stimuli in memory. This stimulus-free delay period is of special interest in neuroimaging studies, because neural responses measured in this time period are thought to reflect WM processes independent of the sensory stimulation itself. During stimuli presentation, the processes of auditory encoding and memory storage are not easily separated, contrary to the delay period where there is no sensory input and the only task is to retain the stimuli in memory and restore inadequately encoded items. A number of studies have found that increased memory load (i.e., increasing the number of items to be remembered) was associated with enhanced alpha power over central and parietal recording sites during the delay period (Jensen et al., 2002; Leiberg et al., 2006; Obleser et al., 2012). Critically, Obleser et al. (2012) recently found that alpha power in the delay period was not only enhanced with an increasing number of to-be-remembered items, but with the acoustic degradation of the items.

In the present study, a version of the Sternberg test modified by Obleser et al. (2012) was applied to investigate the effects of varying memory load and the level of background noise on alpha oscillations measured by electroencephalogram (EEG) recording. We tested older participants with varying degrees of HL. In line with prior studies, we expected decreased task performance with higher memory load and higher levels of background noise. We hypothesized that alpha power would increase with the severity of HL, suggesting that internal auditory degradations increase the load on neural WM mechanisms in speech processing. Furthermore, it was of interest whether such increased expenditure of cognitive resources would reach a limit and break down (i.e., reminiscent of the CRUNCH hypothesis put forward by Reuter-Lorenz and Cappell, 2008) in listeners with the most severe HL and/or under highest task demands (i.e., highest memory load and most severe background noise).

## MATERIALS AND METHODS

### PARTICIPANTS

**FIGURE 1:**
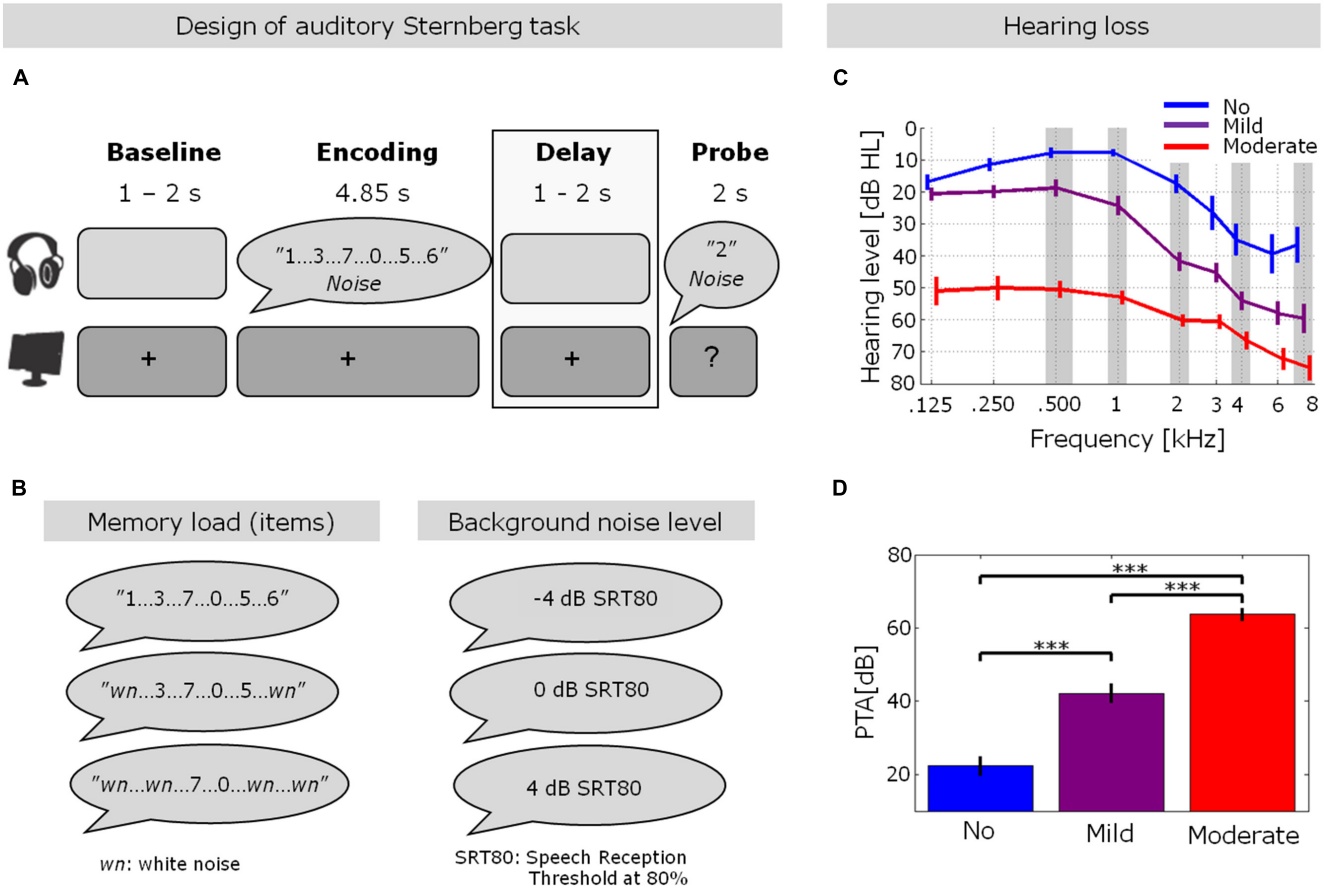
**Hearing thresholds and experimental design. (A,B)** Trial design in the auditory Sternberg task. After an initial silent baseline period, participants were presented with a varying number of spoken digits (2, 4, or 6; see *Experimental procedure* for details) embedded in three different individually adjusted background noise levels (–4, 0, or 4 dB relative to the individual speech reception threshold at 80%, SRT80). After a silent delay period, participants indicated whether a probe digit was presented during the encoding. The gray box highlights the stimulus-free delay period, which was the focus of the EEG data analysis in the present study. **(C)** Pure-tone hearing thresholds for the three hearing loss (HL) groups (blue: no HL, purple: mild HL, red: moderate HL). Error bars indicate ±1 SEM. **(D)** Pure-tone average across frequencies highlighted with gray shading in **(C)** (0.5, 1, 2, 4, and 8 kHz) for the three groups of HL (***p < 0.001; one-way ANOVA with Fisher’s LSD *post hoc* analyses). These PTA values are also shown in **Table 1**. Error bars indicate ±1 SEM. The figure is adapted from Obleser et al. (2012).

Twenty-nine native Swedish speaking participants (16 females, age range: 62–86 years, mean age 72.2 years), recruited from the audiology clinic at the University Hospital of Linköping in Sweden, participated in this study. Participants were recruited to show large inter-individual variability of auditory pure-tone thresholds. Participants were grouped according to their pure-tone average (PTA), across 0.5, 1, 2, 4, and 8 kHz into three groups of HL (no/mild/moderate HL). The hearing threshold at 8 kHz was included in the PTA since sensitivity loss at higher frequencies is known to accompany age-related HL (CHABA, 1988). Separate one-way ANOVAs showed no difference in age between groups (*p* = 0.114), but a significant difference in HL (*p* < 0.001), with Fisher’s Least Significant Difference (LSD) *post hoc* analysis showing significant differences between the three groups (all *p* < 0.001). Participant information is shown in **Table 1** and **Figures 1C,D**.

**Table 1:** Participant information.

	Hearing threshold range [dB HL]	Pure-tone average (PTA) [dB HL]	Age [years]	No. of females
No hearing loss (HL; *n* = 8)	0–25	22.3 (7.1)	68.8 (4.6)	4
Mild HL (*n* = 11)	25–50	42.1 (8.4)	72.5 (5.5)	7
Moderate HL (*n* = 10)	50–80	63.7 (5.2)	74.6 (6.4)	5
Total (*n* = 29)		44.1 (17.9)	72.2 (5.8)	16

Participants grouped according to their HL (no/mild/moderate HL; first column), defined based on three ranges of hearing thresholds (second column). Values in parentheses indicate one standard deviation. Average hearing threshold levels for the three groups across 0.5, 1, 2, 4, and 8 KHz are shown in the third column. Columns four and five list participants’ mean age and number of females in the three groups, respectively. The bottom row shows average data across the entire sample of participants.

Participants all gave informed consent and were given no financial compensation for their participation. The study was approved by the regional ethical review in Linköping, Sweden and conformed with the Helsinki Declaration of Ethical Principles for Medical Research Involving Human Subjects.

### EXPERIMENTAL DESIGN

#### Speech materials

The stimuli consisted of the monosyllabic Swedish digits “0,” “1,” “2,” “3,” “5,” “6,” and “7,” spoken by a female talker and recoded in a soundproof booth at a sampling rate of 22.05 kHz. For a natural co-articulation, the digits were recorded as triplets. The triplets were adjusted to the same root-mean-square (RMS) level, and then the first digit was extracted without silent intervals before and after each waveform, resulting in an average digit duration of 677 ms (SD: 103 ms). The recordings were originally used for the Swedish digit triplets test (Drullman et al., 2005; Larsby et al., 2011).

The final audio files were generated by adding speech-shaped noise to the digits at the individualized SNR levels (see below). Due to the short duration of the spoken digits acceptable speech-shaped noise could not be generated based on the spectrum of the digits. The speech-shaped noise was taken from the Dantale II test, a standardized speech intelligibility test (Wagener et al., 2003). Speech-shaped noise is random stationary broadband noise, with the same long-term average frequency spectrum as natural speech.

#### Stimulus presentation

All participants were wearing Agil hearing aids (Oticon A/S, Smørum, Denmark) with individual quasi-linear amplification. The quasi-linear amplification accounts for the audibility of soft (inaudible speech) sounds by incorporating a fast-acting gain adjustment at the onset of the presented sounds and maintaining this gain throughout the presentation of the sounds with a very slow-acting gain adjustment (for details see Simonsen and Behrens, 2009). No changes were made to the time constant throughout the sound presentation, and the hearing aid amplification can be considered linear, meaning that the hearing aid output intensity increased at the same rate as the intensity of the acoustic input. The noise reduction algorithm and volume control normally available on these hearing aids were disabled during the entire experimental session.

All auditory stimuli were presented directly through the hearing aids using the Direct Audio Input (DAI). The experiment was conducted in an electrically shielded soundproof booth. Visual cues and instructions were presented on a 1280 by 1024 resolution screen, with the participants positioned 1 m from the screen.

#### Individual adjustments of SNR levels

To ensure equal intelligibility of the stimulus materials for all participants despite large inter-individual differences in hearing thresholds (see **Figures 1C,D**; **Table 1**), the background noise levels were individually adjusted. To this end, participants listened to and repeated 40 spoken sentences from the Swedish version of hearing in noise test (HINT; Hällgren et al., 2006). The output presentation level was 70 dB SPL, which was presented through the DAI of the hearing aids and amplified according to the individual audiograms. In an adaptive tracking procedure (Levitt, 1971), we determined the background noise level (measured as the signal to noise ratio between speech and background noise) at which each participant was able to repeat 80% of the words in a sentence. This value for an individual participant will be referred to as the Speech Reception Threshold (SRT) of 80% (denoted 0 dB SRT80). In the Sternberg test, the individual 0 dB SRT80 level was used as the intermediate background noise level for the participant in question. The lower and higher background noise levels were generated by raising or lowering the SNR by 4 dB from the obtained 0 dB SRT80, denoted 4 dB SRT80 and –4 dB SRT80, respectively. To maintain a constant overall intensity level of the stimuli played from the presentation computer at ∼70 dB SPL, both the level of the signal (i.e., the digits) and the level of the background noise were adjusted. For instance, for the 4 dB SRT80 condition, the noise level was lowered by 2 dB in intensity, and the signal level was raised by 2 dB relative to the 0 dB SRT80.

#### Experimental procedure

After the individual adjustment of SNR levels, the actual experiment was performed. An auditory version of the Sternberg paradigm (Sternberg, 1966), inspired by Obleser et al. (2012), was used, employing a 3 × 3 design of the orthogonal factors memory load (2, 4, or 6 digits to be remembered) and background noise level (4 dB, 0 dB, or –4 dB relative to the individual level at which 80% of the words were correctly recalled in noise). Each trial started with the presentation of a central fixation cross for 1–2 s (randomly varied duration), followed by the encoding phase, in which 2, 4, or 6 digits were presented in speech-shaped noise (**Figures 1A,B**). The noise onset always preceded the onset of the first digit by 50 ms to avoid masking of the first digit by the noise onset. In trials with two and four digits, flanking sounds of white noise, at the same intensity level as the spoken digits, were presented to always ensure the presentation of six sounds. The sounds (digits and flanking noises) were presented with an onset-to-onset stimulus interval of 0.8 s, resulting in a total encoding time of 4.85 s, after which the noise was also terminated.

The encoding was followed by a stimulus-free delay period, in which the participants were to retain the presented digits in their memory. The delay phase had a duration of 1–2 s (randomly varied). Lastly, a probe digit was presented in the same background noise level as during the encoding interval. Again, the noise started 50 ms prior to the probe digit. During this 50 ms interval, the fixation cross changed to a question mark, signaling that the participants were to indicate, via a button press on a response box, whether the probe digit appeared in the encoding phase (response window of 2 s). Participants were not instructed to use any particular finger(s) for pressing the response buttons, nor were the button positions varied between participants. If participants required more than 2 s to respond, they were instructed to be faster on the next trial and informed that no response was recorded. Feedback was given after each trial, consisting of either ‘correct,’ ‘incorrect,’ or ‘no answer registered, please answer faster.’ In half of the trials, the probe digit appeared during encoding.

Trials for the nine conditions in the 3 (memory load) × 3 (background noise level) design were presented in 10 blocks. Due to the length of the test, the 10 test blocks were separated into two recordings of five blocks. Each recording lasted ∼45 min with a break of 15 min between the two recordings. Each recording was initiated with a training block of 11–25 trials from all nine conditions. Each test block consisted of a minimum of 18 trials with 2 trials for each condition, presented in a randomized order. The actual number of trials per block was determined by the number of unanswered trials. That is, for each trial in which no answer was registered due to a response time longer than 2 s, an extra trial was added to the block. Overall, 20 trials with registered answers were recorded in each condition for each participant.

### EEG RECORDING AND PREPROCESSING

The EEG was recorded using an EGI system (Electrical Geodesic Inc., Eugene, OR, USA) with 128 Ag/Ag-Cl channels. Six occipital and one central electrode were disconnected from the electrode net and used for other physiological measurements which will not be reported here. The EEG was recorded at a sampling rate of 250 Hz using Cz as the reference. All electrode impedances were maintained below 50 kOhm. The EGI system incorporates analog elliptical high- and low-pass with cut-off frequencies at 0.1 and 125 Hz (the Nyquist frequency), respectively. Filtering was performed before analog-to-digital conversion of the EEG.

Oﬄine, the EEG data were analyzed using customized MATLAB scripts (R2011b, MathWorks Inc.) and the Fieldtrip toolbox (Oostenveld et al., 2011). Trials with response times longer than 2 s were excluded from all further analyses. The data were divided into epochs of sufficient length (–5 to +11 s around the onset of the first digit/flanking noise) to avoid data loss at the edges of the time-frequency representations due to windowing effects. The epoched data were bandpass filtered using an acausal sixth order IIR Butterworth filter between 0.5 and 45 Hz and re-referenced to the average of both mastoids. Before further analyses, 18 electrodes used for recording the electrooculogram (EOG) or positioned on the cheeks and jaw were removed for technical reasons.

Individual channels containing artifacts were identified through visual inspection and repaired by averaging over adjacent electrodes (according to the nearest neighbor approach implemented in the *ft_channelrepair* function in Fieldtrip). Data from one participant from the mild HL group were excluded from all further analyses due to a high number of artifact-contaminated channels. To remove further artifacts, an independent component analysis (ICA) was performed, and components containing eye blinks, saccadic eye movements, muscle activity, and heartbeats were identified by inspection of components’ topographies and time courses and rejected. On average, 22% (SD: 6%) of the components were removed.

The time-frequency representation of oscillatory power in each trial was obtained by convolution of single trial time domain data with a family of Morlet wavelets (width: seven cycles). This analysis was performed for frequencies from 0.5 to 30 Hz in steps of 0.5 Hz and from –5 to +11 s around the onset of the first digit/flanking noise in steps of 0.05 s. Note that this long time interval included the baseline period, encoding, delay, and probe (**Figure 1A**). The power of each time–frequency–electrode bin was calculated for each trial by taking the square norm of the complex wavelet coefficients. Adjustment for inter- and intra-individual variability in oscillatory power was performed by means of subtraction and division by the average power of the first 0.4 to 1 s of the baseline interval (relative change from baseline). For further analyses, each trial was split into the following periods: encoding, 0.4–4.8 s relative to first digit/flanking noise onset; delay, 0.4–1 s relative to the offset of the last digit/flanking noise; and probe, 0.4–1 s relative to probe-digit onset. All time intervals disregard the first 0.4 s as to not include evoked activity after stimulus on- or offset in the analysis.

### STATISTICAL ANALYSES

A main motivation of the present study was to investigate the effect of HL on behavioral performance and alpha oscillations in the auditory Sternberg task. However, HL was confounded by age, as evidenced by a positive Pearson’s correlation between age and PTA (*r* = 0.44, *p* = 0.018). To obtain a measure of HL that was independent of age, we calculated the residualized PTA, quantifying the variation in PTA across participants that could not be explained by age. In detail, the residualized PTA was estimated as the residuals of the linear regression of PTA on age. For the remainder of this paper, we will refer to the *z*-scored residualized PTA as ‘rPTA.’ In all further analyses, rPTA was included as a continuous covariate. Moreover, we considered it likely that brain compensatory mechanisms involved in overcoming the adverse listening conditions would not increase linearly with HL, but drop with more severe HL, especially under high memory load/background noise (see Introduction). To model this negative quadratic (inverted u-shape) relationship between HL and behavioral and brain responses, we additionally included the quadratic term rPTA-squared as a second continuous covariate in all further analyses.

#### Statistical analysis of behavioral data

First, we analyzed to what extent the individual adjustments of SNR levels were dependent on participants’ HL. To evaluate whether individualization was needed, we calculated the Pearson’s correlation between the 0 dB SRT80 value from the HINT and the non-residualized PTA.

In the auditory Sternberg task, response times were measured from the onset of the probe digit until the button press by the participant to indicate whether the probe digit appeared in the encoding. Accuracy was calculated as the percentage of correctly answered trials. Changes in task accuracy and response times as a function of the within-subject factors (memory load and background noise level) and the continuous between-subjects covariates (rPTA and rPTA-squared), were investigated using two separate repeated-measures ANCOVAs. All ANCOVAs showed violation of the assumption of sphericity (Mauchly’s test, all *p* < 0.05), hence the Greenhouse–Geisser corrected *p*-values were calculated and reported for all results. Fisher’s LSD tests were used for all *post hoc* analyses.

**FIGURE 2:**
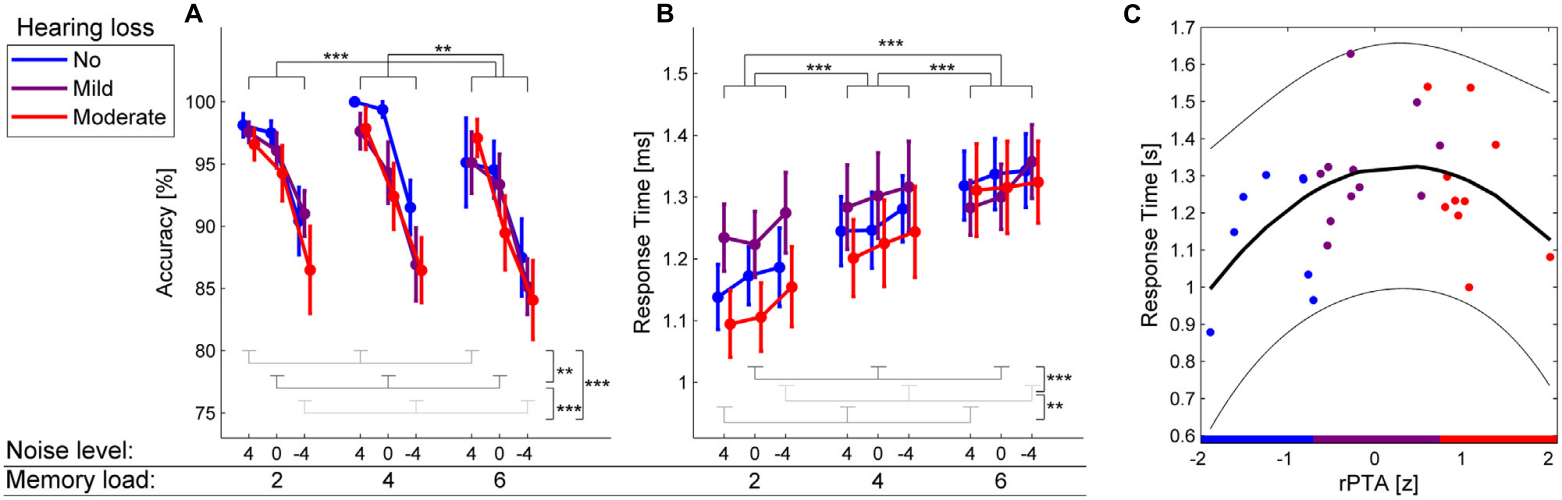
**Behavioral results. (A,B)** Accuracy and response times in the auditory Sternberg task for participants with no HL (blue), mild HL (purple), and moderate HL (red) as a function of memory load (2, 4, 6 to-be-remembered items) and background noise level (4, 0, –4 dB SRT80). Error bars show ±1SEM. ^∗∗^*p* < 0.01, ^∗∗∗^*p* < 0.001. **(C)** Statistically significant quadratic regression between the *z*-scored rPTA and response times (*p* = 0.025). The least-squares regression line is shown in black. The 95% confidence interval is shown in thin lines. The slight overlap in rPTA of the three groups of HL is because the three groups were created before the impact of age on HL was regressed out (see Materials and Methods for details). Note that higher rPTA values indicate more severe HL.

To illustrate the quadratic relationship between rPTA and response times (**Figure 2C**), a quadratic function was fitted to the response time as a function of rPTA using the least-squares approach implemented in the MATLAB functions *polyfit* and *polyval.*

#### Statistical analysis of EEG data

**FIGURE 3:**
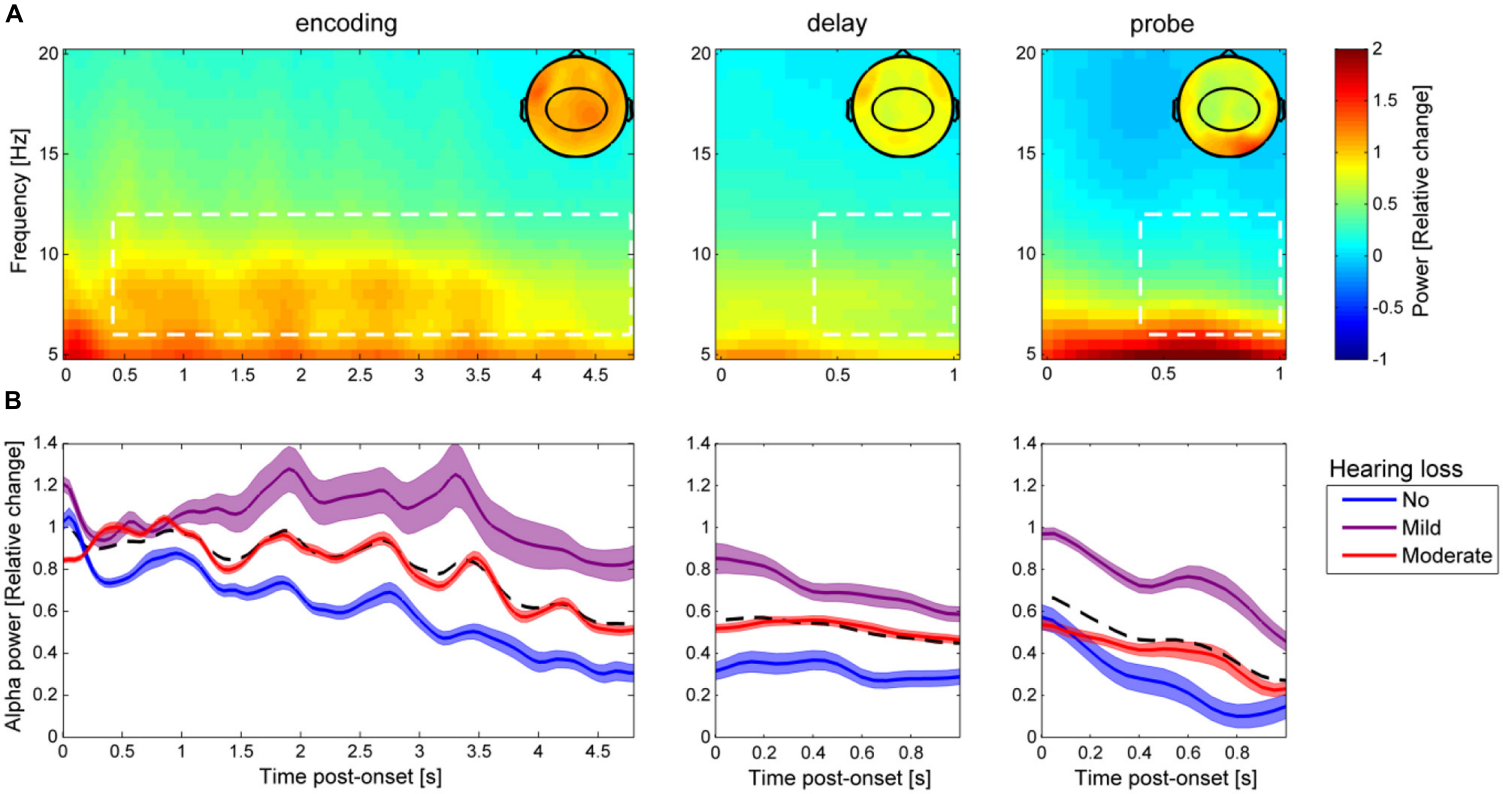
**Alpha power dynamics during the auditory Sternberg task. (A)** Grand-average time-frequency power representation during encoding, delay, and probe (averaged across all participants, in all nine experimental conditions, and for all 31 centro-parietal electrodes highlighted in the topographic maps). The topographic maps show the spatial distribution of alpha power (6–12 Hz) averaged over the time-frequency data highlighted in the white dashed boxes, which were used for statistical analyses. **(B)** The bold lines show average alpha power in the three time periods (encoding, delay, and probe) separately for the three groups of HL (blue: no HL, purple: mild HL, red: moderate HL), with the colored areas indicating ±1 SEM. The black dashed line indicates the average over the three HL groups.

In the analysis of the EEG data, alpha power was averaged across frequencies from 6–12 Hz in a subset of 31 electrodes (**Figure 3A**, topographic maps) and across three time intervals outlined in **Figure 3A**: encoding, 0.4–4.8 s relative to the onset of the first digit/flanking noise; delay, 0.4–1 s relative to the offset of the last digit/flanking noise; and probe, 0.4–1 s relative to the onset of the probe digit. The 31 electrodes were chosen to derive a centro-parietal scalp distribution, which has previously been identified as an important site for alpha activity generation during auditory processing (Krause et al., 1996). Average alpha power during encoding, delay, and probe were subjected to three repeated-measures ANCOVAs with memory load and background noise level as within-subject factors and with rPTA and rPTA-squared as continuous between-subject covariates. All ANCOVAs showed violation of the assumption of sphericity (Mauchly’s test, all *p* < 0.05), hence the Greenhouse–Geisser corrected *p*-values were calculated and reported for all results. All statistical analyses were performed using Statistica (version 12, StatSoft, Tulsa, OK, USA).

**FIGURE 4:**
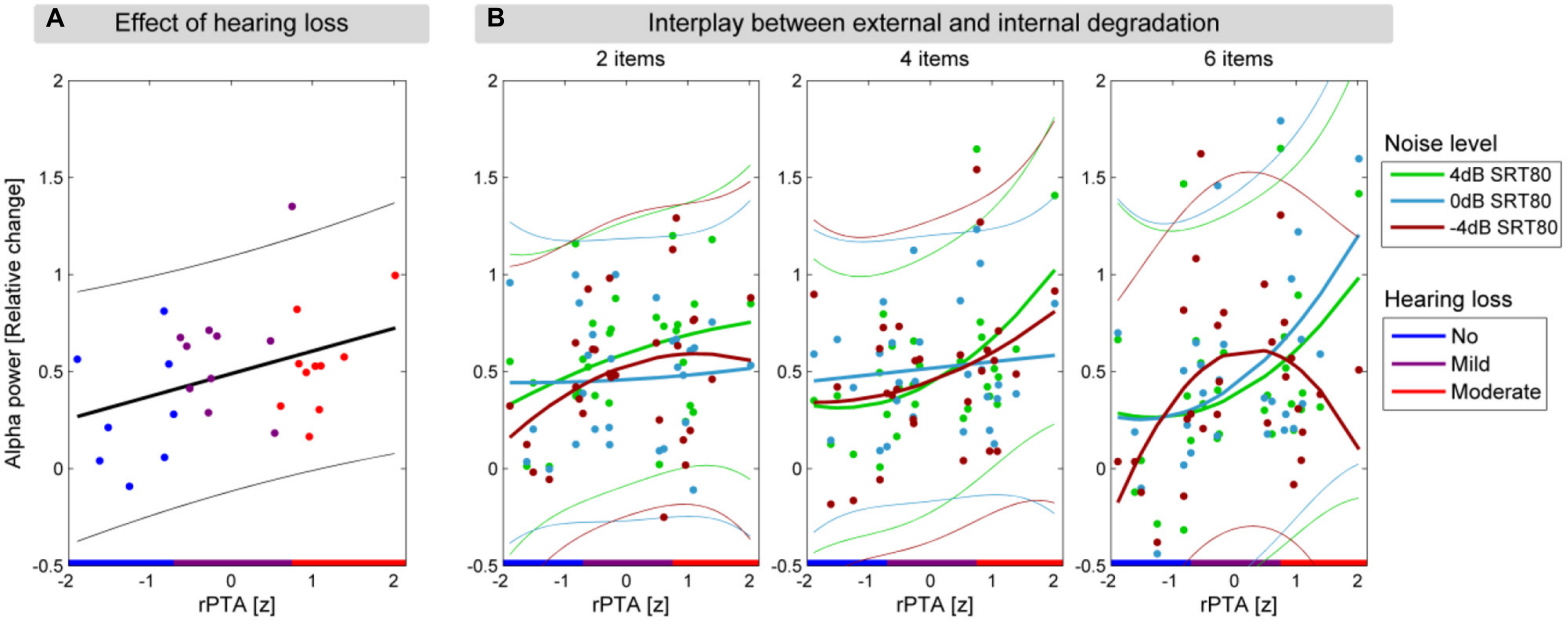
**Hearing loss affects alpha power in the delay period. (A)** The significant linear relationship between alpha power in the delay interval and rPTA (*p* = 0.048). The regression line is shown with a solid black line, and the 95% confidence interval of the regression is shown in thin lines. **(B)** The three panels show the significant interaction between memory load, background noise level, and rPTA-squared, illustrated with quadratic fits between alpha power and rPTA for each background noise level (green: 4, light blue: 0, and dark red: -4 dB SRT80). Each panel shows one of the three memory load conditions (2, 4, and 6 items to be remembered) with alpha power during the delay interval as a function of rPTA with HL groups indicated on the *x*-axis (blue, no HL; purple, mild HL; red, moderate HL).

To illustrate the quadratic relationship between rPTA and alpha power (**Figure 4B**), the fitting procedure described in the section above was applied.

Studies have previously shown an interaction between response time and alpha activity (Klimesch, 2005). Relations between alpha activity during the probe period and response time were therefore evaluated using Pearson’s correlation.

## RESULTS

### INDIVIDUAL ADJUSTMENTS OF SNR LEVELS

The individual adjustments of SNR levels using the SRT80 measure resulted in an average 0 dB SRT80 value of 4.61 dB [standard error of the mean (SEM) = 0.86], meaning that participants on average required an SNR level of 4.61 dB to successfully repeat 80% of words from sentences presented in noise. The 0 dB SRT80 values correlated positively with participants’ non-residualized PTA (*r* = 0.76; *p* < 0.001). This indicates that participants with more severe HL required a higher SNR level of stimulus materials.

### MEMORY LOAD, BACKGROUND NOISE LEVEL, AND HEARING LOSS IMPACT PERFORMANCE

**Figure 2A** shows the average accuracy for the three levels of memory load (2, 4, 6 digits) and the three background noise levels (–4 dB SRT80, 0 dB SRT80, 4 dB SRT80) in the auditory Sternberg task. The main effect of memory load on accuracy was significant [*F*(2,50) = 6.26, *p* = 0.005]. *Post hoc* tests revealed significantly increased accuracy for two compared with six items (*p* < 0.001) and for four compared with six items (*p* = 0.002) but not for two compared with four items (*p* = 0.718). Additionally, the main effect of background noise level on accuracy was significant [*F*(2,50) = 28.35, *p* < 0.001], with the *post hoc* analysis showing a significant decrease in accuracy with increasing noise level (all *p* < 0.01). There were no significant main effects of rPTA [*F*(1,25) = 1.86, *p* = 0.185) or rPTA-squared [*F*(1,25) = 1.94, *p* = 0.176], indicating that the degree of HL by itself did not significantly impact task accuracy. None of the interactions between background noise level, memory load, rPTA, and rPTA-squared were significant (all *p* > 0.195).

**Figure 2B** shows the average response times for the three memory loads and background noise levels. The main effect of memory load on response times was significant [*F*(2,50) = 24.73, *p* < 0.001]. *Post hoc* tests revealed significantly longer response times for six compared with four and two to-be-retained digits, as well as for four compared with two digits (all *p* < 0.001). The main effect of background noise on response times was significant as well [*F*(2,50) = 8.34, *p* = 0.001]. *Post hoc* tests revealed significantly longer response times for the highest background noise level (–4 dB SRT80) compared with the intermediate noise level (0 dB SRT80; *p* < 0.001) and the lowest background noise level (4 dB SRT80; *p* = 0.003). Response times in the four and 0 dB SRT80 conditions did not differ significantly (*p* = 0.328). Interestingly, the main effect of rPTA-squared on response times was significant [*F*(1,25) = 5.69, *p* = 0.025]. This indicated a significant quadratic relationship between response times and the degree of HL in such a way that response times increased from no to mild HL, while response times decreased again for participants with the most severe HL (see **Figure 2C**). Neither the main effect of rPTA [*F*(1,25) = 1.85, *p* = 0.185], nor any interaction between memory load, background noise, rPTA, and rPTA-squared (all *p* ≥ 0.13) reached significance.

### TEMPORAL DYNAMICS OF ALPHA OSCILLATIONS

**Figure 3A** shows the grand-average baseline corrected time-frequency power representation (collapsed over all nine experimental conditions) for all participants throughout the encoding, delay, and probe periods of the auditory Sternberg task. The time course of alpha power (6–12 Hz; averaged over 31 scalp electrodes highlighted in topographic maps) for the three groups of HL are indicated in **Figure 3B**. Descriptively, alpha power decreased over the trial time course from encoding to delay and also during the probe interval. Normal hearing participants (no HL) exhibited the lowest alpha power in encoding, delay and probe, while the mild HL group showed the highest and the moderate HL group exhibited intermediate alpha power.

### Hearing loss affects alpha oscillations under load

We analyzed whether alpha power during the stimulus-free delay interval was dependent on memory load, background noise level, and HL. To this end, the average alpha power (6–12 Hz) across 31 centro-parietal electrodes during the delay interval (0.4–1 s relative to the offset of the background noise) was submitted to a repeated-measures ANCOVA with the factors memory load and background noise level and the continuous covariates rPTA and rPTA-squared. None of the main effects including background noise level [*F*(2,50) = 1.23, *p* = 0.299], memory load [*F*(2,50) = 0.04, *p* = 0.598], or rPTA-squared [*F*(1,25) < 0.01, *p* = 0.989] were significant. Importantly, however, the main effect rPTA was significant [*F*(1,25) = 4.31, *p* = 0.0483], indicating that alpha power during the delay increased significantly with the degree of HL (**Figure 4A**).

Moreover, the two-way interaction background noise level × rPTA-squared [*F*(2,50) = 6.34, *p* = 0.004] as well as the three-way interaction background noise level × rPTA-squared × memory load were significant [*F*(4,100) = 2.86, *p* = 0.042]. The direction of the significant three-way interaction is illustrated in **Figure 4B**. For the two lower memory loads (two and four to-be-remembered items), alpha power during the delay period increased moderately with the degree of HL for all background noise levels. This pattern of results changed significantly under the highest memory load (six to-be-remembered digits); here, alpha power strongly increased with HL under the two more favorable background noise levels (4 and 0 dB SRT80), but under the most severe background noise level (–4 dB SRT 80), alpha power increased only for participants with mild HL, whereas it decreased again for participants with moderate HL. The significant interaction between background noise level and rPTA (*p* = 0.004) is not shown, but resembles the same behavior as observed for six items to be remembered shown in **Figure 4B**. None of the remaining interactions among rPTA, rPTA-squared, memory load, and background noise level were significant (all *p* > 0.15).

The main hypothesis of this experiment was focused on identifying condition and HL effects on alpha power during the delay. However, Obleser et al. (2012) also report smaller condition effects during the encoding and probe period. We therefore investigated alpha power during the encoding (0.4–4.8 s relative to the onset of the first digit/flanking noise) and probe (0.4–1 s relative to probe digit onset) interval as well. For the encoding interval, none of the main effects of memory load, background noise level, rPTA, and rPTA-squared, nor any interactions reached significance (all *p* > 0.14). During the presentation of the probe, a main effect of rPTA-squared was found [*F*(1,25) = 9.63, *p* = 0.004], while no other main effects or interactions were significant (all *p* > 0.12). Notably, an effect of rPTA-squared is also observed on the response time and the relationship between alpha activity during the probe, and the response time was investigated. A Pearson’s correlation showed a positive relationship (*r* = 0.35, *p* = 0.068) between alpha power during the probe and response times, meaning that participants with higher alpha power during the probe interval showed longer response times. A similar relationship was not observed between the alpha power during the delay period and the response times (*r* = 0.15, *p* = 0.42).

## DISCUSSION

In this study, we tested whether HL in older participants had an impact on the neural mechanisms of WM under changing task demands implemented by varying degrees of memory load and background noise. Our main findings can be summarized as follows: first, irrespective of HL, increasing memory load and higher background noise levels led to performance decrements in the auditory Sternberg paradigm. Second, the effects of the increasing memory load and background noise level on alpha activity during the delay were co-determined by the degree of HL. That is, participants suffering from a higher degree of HL exhibited a breakdown in alpha activity with increasing task difficulty, which was not observed for the participants with mild or no HL. These findings show how an internal auditory degradation (i.e., HL) interacts with external acoustic challenges during adverse listening.

### THE EFFECT OF RETAINING AUDITORY STIMULI

Effects of WM processing on alpha power have been often observed only during the retention of stimuli in both auditory (van Dijk et al., 2010; Obleser and Weisz, 2012; Obleser et al., 2012; Becker et al., 2013; Scharinger et al., 2014) and visual tasks (Jensen et al., 2002; Schack and Klimesch, 2002; Sander et al., 2012b). It was therefore not unexpected that modulations of alpha power in this study were also found in the delay period.

The linear main effect of rPTA on alpha power in the delay period (**Figure 4A**) showed that alpha power increases with more severe HL, independent of task difficulty. This linear effect occurs despite the quadratic tendency seen in **Figure 3B**. The linear relationship in **Figure 4A** arose from large individual differences in alpha power, especially in the mildly impaired group, and was also affected by the residualization performed to remove age effects: first, this dependence of alpha power on HL is observed during the retention of the to-be-remembered digits, where no active listening is involved. Second, all participants were wearing hearing aids to equalize audibility of the digits presented during the encoding across participants. Interpreting the alpha activity as a sign of WM involvement (Jensen et al., 2002), our study shows that a higher degree of WM involvement is needed to overcome more severe HL to successfully retain the auditory information. This view of increased WM involvement with increased HL has been put forward in a number of studies (Pichora-Fuller and Singh, 2006; Rönnberg et al., 2008; Shinn-Cunningham and Best, 2008). The Ease of Language Understanding (ELU) model developed by Rönnberg et al. (2008) explains the involvement of the WM in speech understanding under adverse conditions. In detail, the ELU model builds on the ability to match auditory stimuli with a preexisting long-term memory store of phonological representations. When suffering from a HL, this match cannot readily be made due to the internal degradation. Hence WM processes are required for extracting acoustical cues that can trigger a phonological match and ensure a successful understanding. In line with the ELU model, the linear relationship between HL and alpha power can be interpreted as the increased WM resources needed to perform successful phonological matching in listeners with HL. Interestingly, the effect of HL on alpha activity is observed for participants wearing hearing aids, which is thought to ensure equal audibility, but arguably cannot restore the WM resources needed to retain speech stimuli.

Hearing aids can indeed ensure audibility and restore intelligibility in quiet situations, while other aspects of listening, such as processing of temporal cues, are not alleviated by amplification (Ardoint et al., 2010). Furthermore, speech intelligibility in noisy situations also remains affected by HL and cannot be fully restored by amplification (Plomp, 1978; Dillon, 2001). This is indeed evident from the positive relationship between HL and the 0 dB SRT80 value. Peelle et al. (2011) found that increased HL was correlated with decreased gray matter volume of the auditory cortex, i.e., a structural change in the brain. If HL causes structural changes in the auditory cortex, this might explain why individual HL compensation via amplification does not nullify such structural deviation in the auditory system, and HL-dependent effects, such as the present ones, are observed despite hearing aids being employed.

The impact of the experimental conditions (memory load and background noise level) proved only to be significant in interactions with HL. Our results showed that when increasing the external degradation, i.e., the background noise level, an increase in alpha activity with HL was observed for the lower levels of background noise. However, for the highest background noise level, a breakdown in alpha activity was observed for the participants with the most severe degree of HL tested in this study (moderate HL). This breakdown in alpha power is only observed when participants have to remember six digits in the most difficult noise condition (**Figure 4B**). The almost linear increase in alpha power with HL severity observed at lower background noise levels (4 and 0 dB SRT80) suggests that although the noise levels are individualized, participants with increased HL require additional WM resources to be able to perform the task. Indeed, it has previously been suggested that people suffering from HL need to allocate additional resources to process auditory information (Rabbitt, 1991). The findings in this study lend neural support to this hypothesis.

The breakdown in alpha power with increased HL and background noise level further suggests that the participants suffering from reduced hearing reach a ceiling at which no further enhancement in alpha activity can be achieved, and alpha power begins to decrease. Such alpha power breakdown has been observed before when older participants, not considering HL, are subjected to a higher WM load in a visual Sternberg task, while no effect of age was observed on task accuracy (Sander et al., 2012b). Similar findings of neural activity breakdown with high WM loads for increasing age have been observed in fMRI studies (Reuter-Lorenz and Cappell, 2008; Schneider-Garces et al., 2010; Grady, 2012). Also here, the activity breakdown is not necessarily accompanied by changes in task accuracy. According to the “compensation-related utilization of neural circuits” (CRUNCH) hypothesis, the brain increases its activation to engage more neural resources as a result of aging, independent of WM involvement. However, with increasing WM demands, this recruitment reaches a ceiling, and the activity decreases, although no changes in task performance are observed (Reuter-Lorenz and Cappell, 2008). We suggest that, similar to increasing age, more severe HL can cause neural activity breakdown as a result of having to engage more WM resources than participants with better hearing. It is believed that the cause of the observed breakdown is a combination of the two observations that: participants with more severe HL experience generally higher WM involvement (independent of experimental conditions, **Figure 4A**) and during WM tasks they have increased WM involvement (**Figure 4B**). To our knowledge our results are the first to demonstrate a breakdown of neural activity with increased HL.

Alpha power during the delay was affected by memory load in a three-way interaction with background noise and rPTA-squared. Our experimental design was modified from the auditory Sternberg task applied by Obleser et al. (2012), who found main effects of both memory load and auditory degradation (obtained through noise-vocoding of the digits) on alpha activity. The lack of a main effect of memory load in the present study might be explained best by the differences in participants (older hearing impaired vs. younger normal hearing), rather than auditory degradation (background noise vs. noise-vocoding). Both of these changes were introduced to achieve some gain in external validity in the present study.

Although we corrected for the difference in age between participants in this study, we cannot account for the average differences between younger and older persons, which has been proven to affect both alpha activity and WM resources (Klimesch, 1999; Sander et al., 2012a). Although increased age might have resulted in participants having generally less WM resources available and thereby reaching alpha power breakdown, differences in cohort age between the studies cannot explain the non-significant main effect of memory load in the present study. We suggest that the lack of memory load effect can be explained by the fact that the hearing impaired participants are already performing at ceiling and cannot further increase their alpha activity when subjected to higher memory loads and/or background noise levels. This statement is supported by two observations: firstly, that the alpha power increased with HL, independent of the experimental condition. Secondly, that the conditions effects (rPTA × background noise level and rPTA × background noise level × memory load) showed a decrease in alpha power for the moderately impaired participants, c.f. **Figure 4B**.

### NO EFFECTS OF HEARING LOSS ON TASK ACCURACY

To adjust for the differences in HL, the background noise levels were individualized using the SRT80 measure obtained from the HINT test (for details see Materials and Methods). The positive relation between HL and 0 dB SRT80 shows that for participants with more severe HL a lower background noise level (i.e., higher 0 dB SRT80) is needed. This relationship emphasizes the importance of individualizing the background noise level to ensure equal task accuracy across all participants, independent of HL. Indeed, the non-significant effect of HL on task accuracy confirms the success of applying individual noise levels.

As hypothesized, task accuracy significantly decreased both with increased memory load and background noise level. As **Figure 2A** shows, background noise levels showed stronger effects on task accuracy than changes in the memory load. In line with the modulations of alpha activity, this finding emphasizes that auditory degradation induces a larger WM involvement than changes in the memory load for the memory loads and background noise level tested in this study. Significant effects of the experimental conditions on task accuracy have sometimes been reported in auditory and visual Sternberg tasks (Rojas et al., 2000; Jensen et al., 2002; Sander et al., 2012b), but most studies aim at having no condition effects on accuracy (Sternberg, 1966; Lehtelä et al., 1997; Leiberg et al., 2006; Obleser et al., 2012). As noted by Rojas et al. (2000), the confounding effect of task accuracy on response time and alpha activity makes it impossible to determine whether WM processing is indeed involved in solving the task, especially for wrongly answered trials. In this study, effects of memory load and background noise level on task accuracy were found, which is a limitation of the study. However, obtaining task accuracies close to 100% correct for all conditions and participants would require troublesome and time consuming individualization. Alternatively, including only the correctly answered trials in the current analysis would result in an unfeasibly low number of trials per condition. However, as we observe effects of HL on the alpha power, we believe that WM processing was involved during task solving.

The response times were affected both by the experimental conditions (**Figure 2B**) and HL (**Figure 2C**), the latter showing a speed-up in response times with increased HL. As a sign of stimulus retrieval (Sternberg, 1966), it was expected that the response time would show effects of the experimental condition as well as HL. The increase in response times from normal to mildly impaired hearing suggests that increasing internal degradation of the auditory signal results in longer processing times of the probe digit. As HL increases from mild to moderate, participants’ strategy might change resulting in shorter response times (**Figure 2C**).

The effect of rPTA-squared on alpha activity during the probe also proved to be significant and although the correlation between the alpha activity during the probe and the response times only approached significance (*p* = 0.068), we believe that the changes in alpha power during the probe period arise from changes in the speed of information processing (Klimesch, 2005) and not WM processing as such.

In summary, the present findings suggest that despite being compensated for the loss of hearing through hearing aid amplification and by individually setting the administered signal-to-noise ratios, higher degrees of HL are detrimentally affecting a cardinal neural mechanism of overcoming adverse listening conditions, namely the increase in posterior alpha power. Apparently, participants with moderate HL reach a ceiling level at which no more WM resources can be recruited, and thus alpha power begins to decrease again. These findings not only reveal that hearing aid amplification by itself is not sufficient for restoring normal neural signatures of auditory processing, but also suggest that persons suffering from a higher degree of HL reach a WM limit at a lower task demand.

## Conflict of Interest Statement

Eriksholm Research Centre is part of Oticon A/S and as such the salary of Eline Borch Petersen and Thomas Lunner were paid by Oticon A/S. Hearing aids were provided by Oticon A/S.
